# 

**Published:** 1909-10-02

**Authors:** 


					'' l^'SsVoVdEN?B41.fSlOrnc^
Teiecraohic Add ii THE MODERN MEDICAL N fc
I elegraphic Address: AND Telephone Number:
Ospedale, London. || J STITUTIQW AL JOURNAL. 2734 G.rr.rd,
<?> rA<>- ?
HEW SERIES. No. 136, Vol. VI. OkfcbiTWttQ
No. 1208. Vol. XLVII.
Uct. 2nd, 1909.
>-0
PRICE THREEPENCE

				

